# Does age have an impact on surgical and oncologic outcomes after curative resection for gastric cancer: a ten-year single-centre retrospective study

**DOI:** 10.2478/raon-2026-0044

**Published:** 2026-09-07

**Authors:** Matjaz Horvat, Stojan Potrc, Nuhi Arslani

**Affiliations:** Clinical Department of Abdominal and General Surgery, University Medical Centre Maribor, Maribor, Slovenia

**Keywords:** gastric cancer, elderly patients, curative gastrectomy, age-related outcomes

## Abstract

**Background:**

Gastric cancer remains a major health burden in Slovenia, particularly among elderly patients. With increasing life expectancy, a growing number of patients aged ≥ 70 years are expected to undergo curative surgery. This study compared clinical, surgical, and oncologic outcomes between elderly (≥ 70 years) and younger (< 70 years) patients with gastric cancer.

**Patients and methods:**

A single-center retrospective cohort study included patients who underwent curative-intent surgery for gastric adenocarcinoma between 2015 and 2024. Patients were stratified by age. Demographic data, comorbidities, tumor characteristics, surgical variables, postoperative complications (Clavien-Dindo classification), and overall survival (OS) were analyzed. Survival was assessed using the Kaplan-Meier method, and prognostic factors were evaluated using Cox proportional hazards regression.

**Results:**

A total of 433 patients were included (229 < 70 years; 204 ≥ 70 years). Elderly patients had higher American Society of Anesthesiologists (ASA) scores and a higher incidence of major postoperative complications. R0 resection rates and surgical radicality were comparable between groups; however, elderly patients more frequently underwent subtotal gastrectomy and less extensive lymphadenectomy. Perioperative chemotherapy was more commonly administered in younger patients. Tumour characteristics and TNM stage distribution were similar between groups. Five-year OS was significantly lower in elderly patients (37.3% *vs*. 59.6%, p < 0.001).

**Conclusions:**

Curative gastric cancer surgery should not be denied based solely on chronological age. Carefully selected elderly patients can achieve acceptable surgical and oncologic outcomes, highlighting the importance of biological age and functional status in treatment decision-making.

## Introduction

Despite a declining incidence of gastric cancer in Slovenia over the past 60 years, its prevalence remains relatively high, with incidence rates of approximately 26.8 per 100,000 in men and 16.4 per 100,000 in women.^1,2^ Approximately 60% of cases occur in individuals older than 65 years, more frequently in men than in women.^2^ The overall 5-year survival rate for gastric cancer patients in Slovenia is around 28% and has shown little improvement in recent years, largely due to late-stage diagnosis.^3^ According to the Statistical Office of the Republic of Slovenia, life expectancy at birth is approximately 77.7 years for men and 83.8 years for women and continues to increase.^4^ Taken together, these data suggest that the number of elderly patients with gastric cancer will rise in the future.

The European Society for Medical Oncology (ESMO) has developed several resources and guidelines to improve cancer care in elderly patients, including an accepted chronological age cut-off for defining “elderly” patients. Although biological aging is more relevant than chronological age in determining individual health status, chronological age remains a practical and widely used criterion in clinical practice. Currently, 70 years is the most commonly used cut-off in geriatric oncology.^5^ Elderly patients often present with multiple comorbidities that significantly affect treatment strategies, outcomes, and quality of life.^6^ In gastric cancer, delayed diagnosis, comorbidities, and atypical symptom presentation frequently result in more advanced disease at diagnosis in older patients.^7^

Although surgical resection remains the primary curative treatment, elderly patients face increased risks of postoperative complications and mortality.^7^ Furthermore, chemotherapy and targeted therapies require careful consideration due to age-related declines in organ function and a higher risk of treatment-related toxicity.^8^ Nevertheless, several studies have demonstrated that carefully selected, fit elderly patients can benefit from standard treatment approaches, including surgery and systemic therapy, when individualized treatment planning is applied.^8^

The aim of this study was to evaluate differences in clinical, surgical, and oncologic outcomes between elderly (≥ 70 years) and younger patients undergoing curative gastric cancer surgery and to contribute to the existing literature, which has reported inconsistent and sometimes contradictory findings.^9,10^

## Patients and methods

This single-institution retrospective cohort study was conducted at the Clinical Department of Abdominal and General Surgery, University Clinical Centre Maribor, Slovenia, between January 2015 and December 2024. Patients who underwent curative-intent surgery for gastric adenocarcinoma were included. Patients were stratified into two groups according to age (< 70 years *vs*. ≥ 70 years), in line with the criteria proposed by the European Society of Medical Oncology.^5^

Demographic data, clinical findings, histopathological parameters, and clinical outcomes were retrospectively reviewed using a prospectively maintained database. Tumour topography and morphology were coded according to the International Classification of Diseases for Oncology, third edition.^11^ Tumour staging was based on different editions of the Union for International Cancer Control (UICC) TNM classification, with all records subsequently harmonized to the fifth N classification.^12^ Physical status was assessed using the American Society of Anesthesiologists (ASA) classification.^13^ Postoperative complications were graded according to the Clavien-Dindo classification, with complications of grade III or higher included in the analysis.^14^

After surgery, patients were followed every three months during the first two years and every six months thereafter for up to five years. Followup evaluations included physical examination, laboratory tests, ultrasound, and, when indicated, CT, MRI, or endoscopy. Overall survival (OS) was calculated from the date of surgical resection to the date of death, using data from institution-al records and the Cancer Registry of the Republic of Slovenia.

The primary outcome was overall survival according to age group. Secondary outcomes included comparisons of surgical and pathological results and postoperative morbidity between the two groups. Statistical analysis was performed using SPSS version 30 (SPSS Inc., IBM, Chicago, USA). Categorical variables were compared using Fisher’s exact test or the χ^2^ test. Continuous variables were analyzed using Student’s t-test or the Mann–Whitney U test, as appropriate. Independent prognostic factors were identified using Cox proportional hazards regression. Survival curves were estimated using the Kaplan–Meier method and compared with the log-rank test. The study was approved by the Ethics Committee (No: UKC-MB-KME 36/25).

## Results

Between 2015 and 2024, a total of 433 patients underwent potentially curative gastric cancer resection at the University Clinical Centre Maribor. Of these, 229 patients were younger than 70 years and 204 were aged 70 years or older. Baseline demographic characteristics and preoperative clinicopathological features are summarized in Table 1. A significantly higher proportion of women was observed in the elderly group. Elderly patients also had significantly more comorbidities, as reflected by higher ASA scores, which were grouped as < 3 and ≥ 3 for analytical purposes.

**TABLE 1.** Clinicopathological characteristics of the younger and elderly group

		Young (< 70years) N = 229	Elderly (≥ 70 years) N = 204	P - value
		**N (%)**	**N (%)**	
**Sex**	Male	169 (73.8)	130 (63.7)	
	Female	60 (26.2)	74 (36.3)	**0.024**
**ASA**	ASA 1, ASA2	203 (88.6)	127 (62.3)	
	ASA 3, ASA 4	26 (11.4)	77 (37.7)	**< 0.001**
**Tumor site**	Distal third	81 (35.4)	81 (39.7)	
	Middle third	85 (37.1)	87 (42,6)	
	Upper third	62 (27.1)	34 (16.79	
	Whole stomach	1(0.4)	2 (1)	0.069
**Lauren**	Intestinal	94 (48.2)	105 (61.4)	
	Diffuse	39 (20.0)	23 (13.5)	
	Mixed	60 (30.8)	42 (24.6)	0.052
	Missing data	36 (15.7)	34 (16.6)	
**Tumor grading**	0	1 (0.6)	0 (0.0)	
	1	19(11.0)	26 15.0)	
	2	52 (30.1)	59 (30.1)	
	3	100 (57.8	84 (48.6)	0.221
	Missing data	58 (25.3)	35 (17.1)	
**Tumor diameter**	mean	46.3 mm (SD 29.9)	53.2 mm(SD 32.1)	0.418

ASA = American Society of Anesthesiologists physical status classifications system; SD = standard deviation

No significant differences were observed between the groups regarding tumour location. According to the Lauren classification, the intestinal type was more common in elderly patients, although missing data were present in 15.7% of younger and 16.6% of elderly patients. Tumour grading did not differ significantly between groups. Tumour diameter was slightly larger in elderly patients (mean 53.2 mm *vs*. 46.3 mm), but this difference was not statistically significant.

Data on chemotherapy and radiotherapy are presented in Table 2. Significantly more younger patients were candidates for neoadjuvant treatment based on preoperative staging and performance status (135 *vs*. 58 patients). Consequently, all forms of neoadjuvant therapy were more frequently administered in the younger group. Adjuvant therapy was also more common in younger patients (76.9% *vs*. 55.4%), with a proportional distribution between chemotherapy and chemoradiotherapy in both groups.

**TABLE 2.** Operation characteristics and postoperative outcome of the younger and elderly group

		Young (< 70years) N = 229	Elderly (≥ 70 years) N = 204	P - value
		**N (%)**	**N (%)**	
**Type of resection**	Subtotal	38 (16.6)	60 (29.4)	
	Total	132 (57.6)	108 (52.9)	
	Total + distal oesophagecgtomy	41 (17.9)	22 (10.8)	
	Proximal resection	14 (6.1)	10 (4.9)	
	Stump resection	2 (0.9)	3 (1.5)	
	Wedge resection	2 (0.9)	0 (0.0)	
	Stomach + Whipple	0 (0.0)	1 (0.5)	**0.012**
**Splenectomy**	No	187 (81.7)	175 (85.8)	
	Yes	42 (18.3)	29 (14.2)	0.247
**Extent of lymphadenectomy**	D 1	5 (2.2)	16 (7.8)	
	D 1.5	13 (5.7)	16 (7.8)	
	D 2	209 (91.3)	169 (82.8)	
	D 2.5	2 (0.9)	3 (1.5)	**0.028**
**Residual tumour**	R0	225 (98.3)	200 (98.0)	
	R1	2 (0.9)	3 (0.9)	
	R2	2 (0.9)	1 (0.5)	0.178
**Clavien-Dindo complications classification**	< 3b	206 (90.0)	171 (83.8)	
	^3^ 3b	23 (10)	33 (16.2)	**0.04**
**Treatment of complications**	None	176 (76.9)	144 (70.6)	
	Conservative	21 (9.2)	28 (13.7)	
	Interventions US guided, endoscopy	14 (6.1)	7 (3.4)	
	Surgery	18 (7.9)	25 (12.3)	0.1
**Mortality (30 days)**		5 (2.2)	2 (1.0)	0.275
**Mortality (90 days)**		10 (4.4)	17 (8.3)	0.088

Surgical procedures are detailed in Table 3. Elderly patients more frequently underwent subtotal gastrectomy, whereas total gastrectomy with distal esophagectomy was more common in younger patients. Splenectomy rates were similar, but lymphadenectomy was more extensive in younger patients (p = 0.028). R0 resection rates were high and comparable between groups (98.3% *vs*. 98.0%).

**TABLE 3.** Preoperative and postoperative chemotherapy between the groups of younger and elderly patients

		Young (< 70 years) N = 229	Elderly (≥ 70 years) N = 204	P - value
**Neoadjuvant therapy**	Candidate	135 (59.0)	58 (28.4)	
	Not a candidate	94 (41.0)	146 (71.6)	**< 0.001**
**Type of neoadjuvant therapy**	None	94 (41.0)	146 (71.6)	
	Chemo + RT	14 (2.9)	6 (4.6)	
	Chemo - FLOT	74 (32.3)	31 (15.2)	
	Chemo - EOX	47 (20.5)	21 (10.3)	**< 0.001**
**Adjuvant therapy**	Candidate	176 (76.9)	113 (55.4)	
	Not a candidate	53 (23.1)	91 (44.6)	**< 0.001**
**Type of adjuvant therapy**	None	83 (36.2)	138 (67.6)	
	Chemo	121 (52.8)	58 (28.4)	
	Chemo + RT	25 (10.9)	8 (3.9)	**< 0.001**

Chemo = chemotherapy; EOX = epirubicin + oxaliplatin + capecitabin; FLOT = 5-fluorouracil + leucovorin + oxaliplatin + docetaxel; RT = radiotherapy

Postoperative complications were classified as Clavien-Dindo ≥ 3b or lower. Major complications were significantly more common in the elderly group. Conservative management was the most frequent approach in both groups. Interventional procedures were more common in younger patients, while reoperations were more frequent in elderly patients, although these differences were not statistically significant. Ninety-day postoperative mortality was higher in elderly patients but did not reach statistical significance.

Histopathological findings are summarized in Table 4. No significant differences were observed regarding lymphocyte infiltration, vascular invasion, tumour thrombus, extranodal infiltration, or perineural invasion. Some missing pathological data were noted. TNM stage distribution was similar between groups, with T3 being the most frequent stage. Nodal involvement did not differ significantly. Intraoperative metastases were detected in 4.4% of younger and 6.4% of elderly patients.

**TABLE 4.** Histopathological findings between younger and elderly patients

		Young (< 70years) N = 229	Elderly (≥ 70 years) N = 204	P - value
		**N (%)**	**N (%)**	
**Lymphocyte infiltration**	None	68 (32.7)	60 (32.8)	
	Mild	74 (35.6)	71 (38.8)	
	Moderate	56 (26.9)	39 (21.3)	
	Severe	10 (4.8)	13 (7.1)	0.492
	Missing data	21 (9.1)	21 (10.2)	
**Vascular invasion**	None	173 (77.9)	138 (71.9)	
	Present	49 (22.1)	54 (28.1)	0.155
	Missing data	8 (3.4)	12 (5.8)	
**Tumour thrombus**	None	184 (94.8)	162 (91.0)	
	Present	10 (5.2)	16 (9.0)	0.160
	Missing data	35 (15.2)	26 (12.7)	
**Extranodular infiltration**	None	185 (93.4)	159 (87.8)	
	Present	13 (6.6)	22 (12.2)	0.060
	Missing data	31 8 (13.5)	23 (11.27)	
**Perineura invasion**	None	112 (49.8)	86 (43.4)	
	Present	113 (50.2)	111 (56.1)	0.257
	Missing data	4 (1.7)	7 (3.43)	
**T stage**	T0	6 (2.6)	6 (2.9)	
	Tx	0 (0.0)	1 (0.6)	
	Tis	32 14.0)	13 (6.4)	
	T1a	11 (4.8)	10 (4.9)	
	T1b	10 (4.4)	12 (5.9)	
	T2	23 (10.0)	23 (11.3)	
	T3	91 (39.7)	75 (36.8)	
	T4a	44 (19.2)	55 (27.0)	
	T4b	12 (5.2)	9 (4.49	0.200
**Nodes**	N0	110 (48.0)	88 (43.1)	
	N1	33 (14.4)	26 (12.7)	
	N2	39 (17.0)	37 (18.1)	
	N3a	27 (11.8)	23 (11.3)	
	N3b	20 (8.7)	30 (14.7)	0.377
	Negative nodes	110(48.0)	88 (43.1)	
	Positive nodes	119 (52.0)	116 (56.9)	0.307
**Metastases**	None	219 (95.6)	191 (93.6)	
	Present	10 (4.4)	13 (6.4)	0.353

**FIGURE 1.**
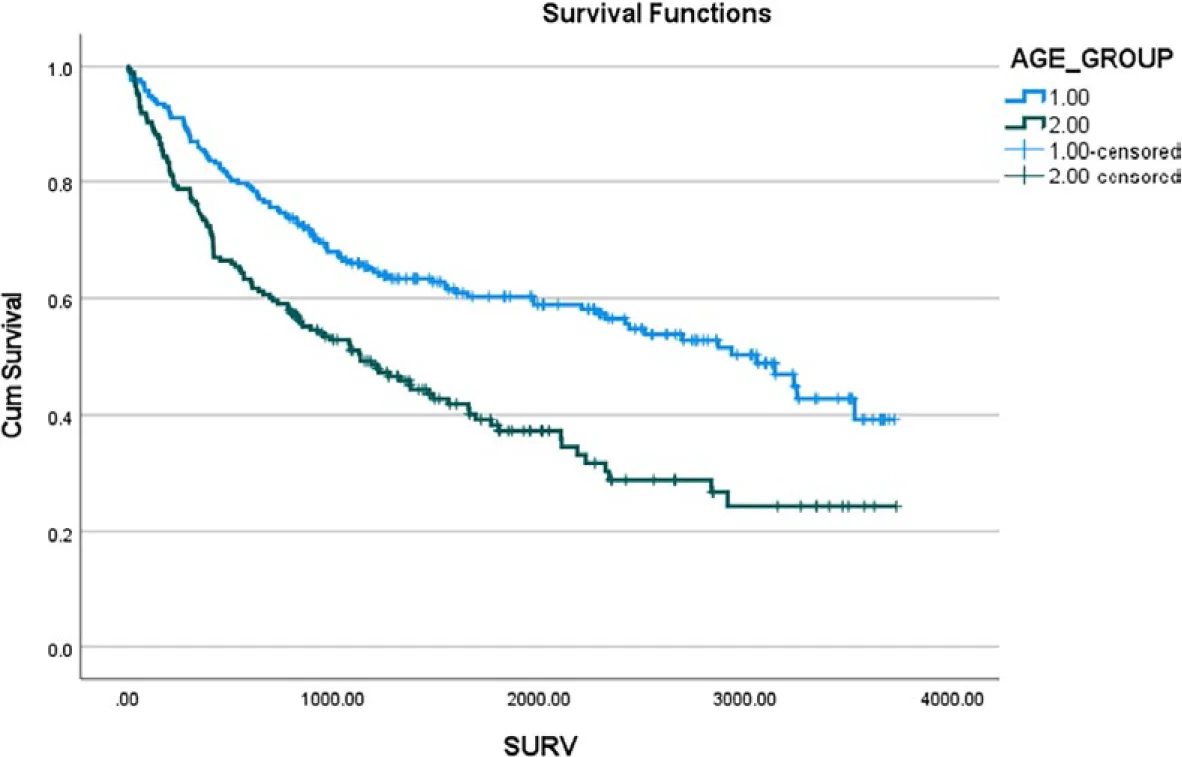
Overall survival (OS) between the younger and elderly group of patients. 1.00 represents patients < 70, 2.00 represents patients > 70.

Overall survival data are presented in Figure 1. Five-year OS was significantly higher in younger patients (59.6%) than in elderly patients (37.3%; p < 0.001).

## Discussion

Age alone should not preclude patients from undergoing curative surgery. In this ten-year retrospective study of potentially curative gastric cancer resections, we compared surgical, pathological, and oncological outcomes between younger and elderly patients. The incidence of gastric cancer was higher in men across all age groups. Notably, the male-to-female ratio increased with advancing age, particularly after menopause, suggesting a potential protective role of oestrogen in premenopausal women.^15^ In our cohort, women accounted for one quarter of cases in the younger group compared with one third in the elderly group (p = 0.024). This finding may also reflect the generally longer life expectancy of women.

Previous studies have demonstrated that higher ASA scores – particularly classes III and IV – are associated with increased postoperative complications and reduced overall survival in elderly gastric cancer patients. A retrospective study of 166 patients aged 80 years or older identified ASA class III–IV as an independent risk factor for postoperative mortality.^16^ Similarly, Wakahara *et al*. reported a significantly higher incidence of postoperative cardiovascular complications among patients with ASA class III or IV, underscoring the predictive value of ASA for systemic postoperative risk. In our study, ASA class III–IV was significantly more frequent in the elderly group (37.7%) than in the younger group (11.4%). This difference was associated with a higher incidence of Clavien-Dindo grade ≥ 3b complications. However, no significant differences were observed in 30- or 90-day perioperative mortality, possibly due to the relatively younger age of our elderly cohort compared with those in previous studies.^17^

Tumour histology and biological behaviour vary with age. A large meta-analysis by Petrelli *et al*., including over 60,000 patients, demonstrated that the intestinal histological type is more prevalent in elderly patients and is generally associated with better differentiation and prognosis, whereas the diffuse type is more common in younger patients and is linked to poorer differentiation. Nevertheless, in elderly patients with early gastric cancer, cancer-specific survival does not appear to differ significantly between intestinal and diffuse types.^18^ Yang *et al*. identified age, tumor location, differentiation, and tumour size as key predictors of overall survival in elderly patients.^19^

In our study, the middle third of the stomach was the most common tumour location in both groups. Tumour location in younger patients was relatively evenly distributed across all gastric thirds, whereas elderly patients showed a tendency toward distal tumour localization, although this difference was not statistically significant. Lauren classification in our cohort was consistent with published data, with the intestinal type accounting for approximately two thirds of cases in the elderly group. However, no significant differences were observed in tumour grading between groups. Although tumour size has been variably reported as a prognostic factor in the literature often losing significance in multivariate analyses unless lymph node evaluation is inadequate.^20-22^ We found no statistically significant difference in tumour diameter, despite slightly larger tumours in the elderly group.

Surgical strategies also differed between age groups. Elderly patients more frequently underwent subtotal gastrectomy, whereas younger patients were more likely to receive total gastrectomy. In the CRITICS trial, subtotal gastrectomy was performed in 45% of patients aged ≥ 70 years compared with 39% of younger patients.^23^ Similarly, in our cohort, subtotal gastrectomy was significantly more common in elderly patients (29.4% *vs*. 16.6%), despite no significant difference in tumor location. Total gastrectomy remained the most frequently performed procedure in both groups. Younger patients also underwent more extensive lymphadenectomy, with D2 dissection performed in over 90% of younger patients and over 80% of elderly patients. This difference likely reflects concerns regarding the higher risk of postoperative morbidity associated with extensive dissections in elderly patients.

Postoperative complication profiles differed between age groups when classified according to the Clavien-Dindo system. While several studies report that minor complications (grade < 3b) occur more frequently in elderly patients, the incidence of severe complications is generally comparable between age groups.^24-26^ In our study, minor complications were similarly distributed (90% in the younger group *vs*. 83.3% in the elderly group). However, severe complications (grade ≥ 3b) were significantly more frequent among elderly patients (16.2% *vs*. 10%). Management strategies were comparable across groups, with conservative treatment being the most common approach. Perioperative mortality was higher in elderly patients, particularly at 90 days, although this difference did not reach statistical significance.

Significant differences were also observed in the use of systemic therapy. Younger patients were more frequently candidates for neoadjuvant chemotherapy or chemoradiotherapy than elderly patients (59.0% *vs*. 28.4%). Given the comparable tumor characteristics between groups, this discrepancy likely reflects differences in comorbidities, functional status, and treatment tolerance. FLOT was the most commonly used neoadjuvant regimen in both groups. Prior studies have shown increased toxicity and impaired quality of life in elderly patients receiving intensive chemo-therapy, emphasizing the importance of careful patient selection.^23,27^ Regarding adjuvant therapy, younger patients were more likely to receive treatment (76.9% *vs*. 55.4%), although TNM staging did not differ significantly between groups. Evidence suggests that the survival benefit of adjuvant chemotherapy is most pronounced in stage III disease, and monotherapy may offer a favourable balance between efficacy and tolerability in elderly patients.^28^

Pathological features such as lymphocytic infiltration, vascular invasion, tumor thrombus, extranodal extension, and perineural invasion did not differ significantly between groups. Nonetheless, previous studies have reported higher rates of vascular and lymphovascular invasion in patients aged ≥ 60 years.^29^ Perivascular invasion is associated with aggressive tumor behavior, poorer prognosis, and significant correlations with histological subtype.^30,31^

As expected, overall survival was shorter in the elderly group, in agreement with existing clearly published data. Puhr *et al*. demonstrated similar stage-specific survival distributions across age groups.^32-34^ Given the comparable tumor characteristics in our study, the reduced overall survival in elderly patients may primarily reflect a higher burden of comorbidities and shorter life expectancy. Importantly, several authors have reported comparable overall survival between younger and elderly patients when curative resection is achieved, supporting the principle that age alone should not be a contraindication to curative surgery.^35^

This study is limited by its retrospective design, with inherent risks of selection bias and inability to infer causality. Reliance on medical records introduces potential inaccuracies due to incomplete or inconsistently documented data. Missing data for key clinical variables may have introduced information bias and reduced statistical power, particularly in subgroup analyses. Finally, the singlecenter nature of the study limits external validity, as institutional practices and patient characteristics may not be generalizable to other settings. Prospective, multi-center studies with standardized data collection are needed to confirm these findings.

## Conclusions

This ten-year retrospective study confirms that chronological age alone should not be considered a contraindication to curative surgery for gastric cancer. Although elderly patients presented with higher ASA scores and experienced more severe postoperative complications, perioperative mortality and oncologic outcomes were comparable when curative resection was achieved. Tumor characteristics and stage distribution were similar across age groups, and standard oncologic principles were maintained in selected elderly patients. The reduced overall survival observed in elderly patients appears to be driven primarily by comorbidities and limited life expectancy rather than tumor-related factors. These findings support an individualized treatment approach based on biological age, functional status, and comorbidity burden rather than chronological age. As the population continues to age, multidisciplinary assessment and tailored treatment strategies will be increasingly important.
